# Green Bio-Assisted Synthesis, Characterization and Biological Evaluation of Biocompatible ZnO NPs Synthesized from Different Tissues of Milk Thistle (*Silybum marianum*)

**DOI:** 10.3390/nano9081171

**Published:** 2019-08-16

**Authors:** Bilal Haider Abbasi, Muzamil Shah, Syed Salman Hashmi, Munazza Nazir, Sania Naz, Waqar Ahmad, Inam Ullah Khan, Christophe Hano

**Affiliations:** 1 Department of Biotechnology, Quaid I Azam University, Islamabad 45320, Pakistan; 2 Department of Botany, University of Azad Jammu & Kashmir, Muzaffarabad 13230, Pakistan; 3 Department of Pharmacy, Quaid I Azam University, Islamabad 45320, Pakistan; 4 Department of Biochemistry, Quaid I Azam University, Islamabad 45320, Pakistan; 5 Laboratoire de Biologie des Ligneux et des Grandes Cultures (LBLGC), INRA USC1328, Université d’Orléans, F28000 Chartres, France

**Keywords:** Zinc Oxide (ZnO), Nanoparticles (NPs), characterization, *Silybum marianum*, in vitro plantlets, cytotoxic assay, green synthesis

## Abstract

The purpose of the current study was green synthesis of ZnO-nanoparticles (NPs) from different tissues of *Silybum marianum* (L.) Gaernt. (i.e., seeds, wild plant, in vitro derived plantlets and callus cultures) followed by extensive characterization and evaluation of their biological potency. ZnO-NPs thus synthesized were subjected to characterization using standard techniques such as XRD, FTIR and SEM. Thermal stability of synthesized NPs was also evaluated using thermo-gravimetric analysis. Highly stable crystalline NPs with size ranging between 30.8 and 46.0 nm were obtained from different tissues of *S. marianum*. These NPs have revealed a wide range of biological applications showing antioxidant, moderate α-amylase inhibitor, antibacterial and cytotoxic potencies. The highest antibacterial activity (20 ± 0.98 mm) was shown by seed extract-mediated ZnO NPs against *Staphylococcus aureus* (ATCC-6538). Seed extract-mediated ZnO NPs also showed the most potent antioxidant activity (27.7 ± 0.9 µgAAE/mg, 23.8 ± 0.7 µgAAE/mg and 12.7 ± 1.9% total antioxidant capacity (TAC), total reducing power (TRP) and DPPH-free radical scavenging assay (FRSA), respectively). All of the synthesized ZnO NPs also showed cytotoxic activity against the hepato-cellular carcinoma (HepG2) human cells. Interestingly, these ZnO NPs were also highly biocompatible, as evidenced by the brine shrimp lethality and human red blood cells hemolytic assays. Among all of the NPs synthesized and used, the effect of seed extract-mediated NPs was found to be most promising for future applications.

## 1. Introduction

One of the most promising areas of research that has emerged in science and technology is “Nanotechnology”, which has introduced a global paradigm shift in life sciences [1]. Nanotechnology has brought about revolutionary transformation in domains ranging from health to industrial materials. The key product of nanotechnology, i.e., nanoparticles (NPs) can be defined as materials that possess at least a unit dimension in the nanometer range (1–100 nm) [2]. In order to formulate these NPs, the biological route has received much more attention due to several important attributes such as safety, environmentally friendly protocols with non-toxic byproducts, gentle reaction condition requirements and the use of natural capping and reducing agents [3]. The NPs thus produced through the biological approach are comparatively stable and safer, with much more diversity in terms of size and shape [4]. The most abundantly used biological route for NPs synthesis is the use of plant extracts for the formulation of ecofriendly NPs, which cuts out the use of noxious chemicals with toxic effects [5]. Plant-mediated NPs synthesis results in the formulation of NPs having definite shape and size. Researchers are also working on the use of the aqueous medium containing hydrolytic reagents for possible capping and stabilization of materials in the nanodimension [6].

The interdisciplinary link between nanotechnology and biotechnology gives rise to a new discipline, namely nanobiotechnology, which specifically deals with production, enhanced potential and utilization of material at the nanoscale for advanced research [7]. The focus of nanobiotechnology is to promote the use of economical and environmentally benign reducing and capping agents obtained from living material such as fungi, microbes, algae and plant material (including tissues, seeds and fruits, etc.) for their feasible use in medicinal compounds, cosmetic industries and water disinfection [8]. The adverse effects of NPs are neutralized due to the presence of natural metabolites in plants and other biological materials [9]. NPs produced as a result of green approaches are the center of consideration in various fields including synthetic chemistry, biotechnology, environmental sciences and biochemistry.

Zinc oxide (ZnO) biosynthesized nanocomposites have unique features. A number of researchers have found it attractive for research in multiple research disciplines due to the novel applications. Some of the important features of ZnO includes piezoelectric, catalytic pyroelectric, optoelectronics and semiconducting capacity [10]. It is an n-type semi-conductor having exciton binding energy of 60 meV, and its energy of band gap at the normal room temperature is 3.37 eV. The nanostructured form of ZnO further adds to its physio-chemical properties due to the increased ratio of surface-to-volume. In order to bring ZnO particles into the nanoscale, a variety of protocols can be utilized which includes the vapor–liquid–solid (VLS) [11], hydrothermal synthesis [12], vapor phase deposition [13], chemical vapor deposition [14], metallo-organic deposition of chemical vapors [15] and zinc oxidation [16]. ZnO NPs with several different morphologies including NPs [17], nanorods [18], nanowire [19], nanobelts [20], nanoflowers [21] and nanoflakes [22] have been synthesized using the aforementioned techniques. Such conventional techniques have several limitations which make their use questionable, and hence, green synthesis has proven an effective alternate for the synthesis of ZnO NPs in order to overcome the issues mentioned earlier [23,24,25].

Several NPs have been evaluated for their potential applications out of which Zinc Oxide (ZnO) NPs have proved to be a vital alternative for remediation of pollutants in the environment, owing to their high absorption capacity for UV rays. ZnO NPs have also been reported to result in elevated production of reactive oxygen species (ROS), including hydrogen peroxides [6,26,27]. Their applications in the semiconductors industry for the production of solar cells are also well documented [28,29,30]. The distinctive photochemical, antifungal, antibacterial, catalytic and UV-filtering potential of ZnO NPs has brought them into the limelight for interdisciplinary research [31]. ZnO NPs have also shown great promise in solar cell industries, displays, bio-sensors, photo-catalysis, pharmaceutical and cosmetic industries [32,33,34]. A variety of plant species have been efficiently used for the reduction and capping of ZnO NPs including *Aloe vera* [7,35], *Calotropis gigantea* [36], *Citrus aurantifolia* fruits [37], *Coriandrum sativum* [38], *Parthenium hysterophorus* L. [39] or milky latex of *Calotropis procera* [40].

The current research focuses on ZnO NPs phyto-synthesis using various extracts of *Silybum marianum* (L.) Gaernt. (Milk thistle) (seeds, wild plant, in vitro derived callus and plantlets). Different biological assays, including antioxidant, antibacterial, antiproliferative assays, together with brine shrimp lethality and human red blood cells hemolytic assays, were conducted to test the potency of synthesized NPs and study whether the different bioresources of the same plant have any impact on NPs synthesis, morphology, size and the ZnO NPs activity.

## 2. Materials and Methods

### 2.1. Seeds Collection

*Silybum marianum* (L.) Gaernt. (Milk thistle) seeds plants were collected from Khyber Pukhtoonkhawa, Peshawar, KP. The wild plants used in the study were sampled from Quaid-i-Azam University, Islamabad.

### 2.2. Reagents

All of the chemicals used in the present study, and in particular, the zinc acetate dihydrate, were obtained from the Sigma-Aldrich Company (Sigma-Aldrich, Saint-Quentin Fallavier, France).

### 2.3. Preparation of Plant Extract

Plant extract was prepared by mixing the respective plant material (seeds, wild plant in vitro derived plantlets and callus) with distilled water in a 1:10 ratio into an Erlenmeyer flask containing 15 g of plant material for 150 mL distilled water. The callus for biosynthesis of ZnO NPs was obtained by inoculating the explant at a hormonal concentration of 0.5 mg/L BAP (6-benzylaminopurine) and 1 mg/L of 1-NAA (1-naphthaleneacetic acid) to induce callus culture. Harvesting of the callus was done after 28 days. The seeds were grinded while the rest of the plant materials were chopped into fine pieces prior to mixing. The resulting mixtures, in the case of seeds, wild plant and in vitro derived plantlets, were boiled for 2 h at 100 °C. The boiled mixture was then incubated at 40 °C overnight. The flask containing callus was placed in a shaker incubator for 24 h instead of heating. The extracts were then filtered and kept in a refrigerator prior to their use.

### 2.4. ZnO NPs Biosynthesis

ZnO NPs biosynthesis was carried out by adding zinc acetate di-hydrate (molecular weight: 219.51 g/mol) to the plant extract in a 100:1 ratio comprising 1 mM of zinc acetate di-hydrate into 100 mL of plant extract. The pH was set at 12 and the extract was then kept for 24 h at 37 °C. After 24 h, ZnO NPs gathered at the base of the Erlenmeyer flask. The supernatant was discarded and the solution containing NPs was introduced into 1.5 mL microcentrifuge tubes and washed with 1 mL of pure ethanol keeping centrifuge for 10 min at 12,000 rpm. The supernatant was disposed of and the left-over pellets were washed thrice with the distilled water. Calcination (500 °C) of the NPs was done for 2 h to increase the crystallinity of the NPs.

### 2.5. Characterization

To confirm the biosynthesis and crystallinity of the ZnO NPs, they were subjected to X-ray Diffraction (XRD) (Model-D8 Advance, Bruker, Mannheim, Germany).

For size and morphological analysis, scanning electron microscopy (SEM) was carried out by using JEOL-JSM-6490LA SEM (JEOL, Tokyo, Japan). Instrument works at 20 kV with 2838 counts per second.

Fourier transform infrared (FTIR) spectroscopy (SHIMADZU 8100 M FTIR, Shimadzu, Kyoto, Japan) was done to study the presence of bio-molecules as capping agents on ZnO NPs surface in the range of 400–4000 cm^−1^.

For determination of thermal stability, thermo-gravimetric analysis (TGA) was conducted. The samples were studied at a temperature ranging from 0 to 1100 °C under a nitrogen atmosphere with an increase of 10 °C/min using TGA (TGA/DSC1, Star e System, Mettler Toledo, Giessen, Germany).

### 2.6. MTT Assay

#### 2.6.1. Cell Culture

Human hepato-cellular carcinoma cells HepG2 (ATCC HB-8065; American Type Culture Collection, Manassas, VA, USA) were cultured in Dulbecco’s Modified Eagle Medium (DMEM) comprising of 10% fetal calf serum (FCS), supplemented with 2 mM l-glutamine, 100 U/mL penicillin, 100 μg/mL streptomycin, 1 mM Na-pyruvate at 37 °C in a humidified 5% atmosphere of CO_2_. Cell harvesting was carried out using 0.5 mM trypsin/EDTA for 1 min at room temperature (25 ± 1 °C).

#### 2.6.2. Evaluation of Mitochondrial Function using Cytotoxicity Analysis: MTT Assay

MTT (3-(4,5-dimethylthiazolyl-2)-2,5-diphenyltetrazolium bromide), a tetrazolium dye was used to access cytotoxic potential of extracts/NPs in vitro. In living cells, MTT is reduced into its insoluble purple product formazan which is measured spectrophotometrically.

In a 96-well plate, pre-seeded HepG2 cells (>90% viability; 1 × 10^4^ cells/well; 200 µL per well) were treated with 200 µg/mL of test samples for 24 h. Later, 10 µL of MTT dye (5 mg/mL) was added per well, followed by incubation of 3 h. Insoluble formazan was then dissolved by adding 10% acidified sodium dodecyl sulfate (SDS). Cells were then incubated overnight. Plates were analyzed at 570 nm using a microplate reader (Platos R, 496. AMP, AMEDA Labordiagnostik GmbH, Graz, Austria).

Untreated HepG2 cells (NTC) were included as controls. DMSO was used as negative control for plant extracts. NPs were sonicated (ultrasonic bath USC1200TH, Prolabo, Sion, Switzerland) prior to cytotoxicity screening and the extracts were centrifuged prior to cytotoxicity screening percent (%) viability being quantified with respect to the NTC sample, using the following equation:

$$\mathrm{Viability}\;(\%)\;=\;\frac{{\mathrm{Sample}}^{’}s\;\mathrm{Absorbance}\;-{\;\mathrm{Control}}^{’}s\;\mathrm{Absorbance}}{{\mathrm{NTC}}^{’}s\;\mathrm{Absorbance}\;-{\;\mathrm{Media}}^{’}s\;\mathrm{Absorbance}\;}\times 100$$

where Abs. of NTC and Abs. of sample corresponds to the optical density at 570 nm respectively for the untreated control samples and treated control samples. Abs of blank and Abs. of sample control corresponds to background optical density. This was quantified in media samples and NPs/extracts only.

For each sample, the experiment was performed twice with three replicates.

### 2.7. Antibacterial Potency

Synthesized NPs were evaluated for their antibacterial potency through disc diffusion assay according to established protocol with slight modification [41,42]. Refreshed bacterial cultures of gram positive, i.e., *Staphylococcus aureus* (ATCC-6538) and *Bacillus subtilis* (ATCC-6633) bacterial strains, and gram negative, i.e., *Escherichia coli* (ATCC-25922), *Pseudomonas aeruginosa* (ATCC-15442) and *Klebsiella pneumonia* (ATCC-1705) bacterial strains, with pre-adjusted seeding density, were prepared. A total of 5 μL of each sample was introduced on sterile disks placed on specific sites on agar seeded plates. Cefixime and roxithromycin acted as a positive control, while DMSO was used as a negative control. The plates were then incubated for a period of 24 h at 37 °C. Following incubation, the zones of inhibition for all of the test samples were measured and recorded. Test samples showing a zone of inhibition ≥12 mm were scrutinized to ascertain MIC (minimum inhibitory concentration) using the standard three-fold micro-broth dilution.

Stock solutions (20 mg/mL in DMSO) of each of the synthesized ZnO nanoparticle was diluted serially in a 96-well microtiter plate (200 µL per well) with Mueller Hinton broth to reach the range of concentration from 3.70 μg/mL up to 100 μg/mL. A standardized inoculum for each bacterial strain was prepared in order to permit the size of inoculum equaling 5 × 10^6^ CFU/mL (colony forming unit) approximately in each well. The plates prepared were then incubated overnight at 37 °C. After completion of incubation period, the MIC was evaluated as the lowest concentration of the test extract inhibiting the bacterial strain growth by measuring optical density (OD) at 630 nm, using a microplate reader (Platos R, 496. AMP, AMEDA Labordiagnostik GmbH, Graz, Austria). All of the test NPs were evaluated in triplicate.

### 2.8. Total Antioxidant Capacity (TAC)

The assessment of the total antioxidant capacity of the NPs was carried out using a phosphomolybdenum-based assay [42,43]. The test sample (4 mg/mL dissolved in DMSO) and DMSO (negative control) amounting 100 μL were separately added to 900 μL reagent containing sulfuric acid, ammonium molybdate and sodium phosphate having concentrations of 0.6 M, 4 mM and 28 mM, respectively. The resulting mixture was subjected to incubation in a water bath for 90 min at 95 °C so as to complete the reaction. The absorbance of standard and test solutions was recorded at 695 nm once it cooled down via PDA spectrophotometer (8354 Agilent Technologies, Waldbronn, Germany). For a blank reading, DMSO was used. The same experiment for the total antioxidant capacity (TAC) was done in triplicate. The antioxidant activity evaluated corresponds to μg/mg dry weight of ascorbic acid equivalent antioxidant capacity (μg AAE/mg DW).

### 2.9. Total Reducing Power (TRP)

To calculate the reducing power of different NPs, potassium ferricyanide colorimetric assay was used [41,42,43]. A total of 400 μL of ferricyanide (1%) in 0.2 mol/L phosphate buffer (pH 6.6) and 100 μL of test sample (4 mg/mL dissolved in DMSO) were combined. The resulting mixture was then incubated at 50 °C for 20 min. A total of 400 μL of trichloroacetic acid (10%) was added to each sample after incubation. The resulting mixture was then centrifuged at room temperature for 10 min at 3000 rpm. A total of 500 μL of the supernatant was collected, to which 100 μL of 0.1% FeCl_3_ and 500 μL of distilled water were added. Finally, optical density at 700 nm was recorded using a microplate reader (Platos R, 496. AMP, AMEDA Labordiagnostik GmbH, Graz, Austria). DMSO alone served as the blank (negative control) whereas ascorbic acid served as positive control. The reducing power of the test samples corresponds to μg/mg dry weight of ascorbic acid equivalent antioxidant capacity (μg AAE/mg DW).

### 2.10. Free Radical Scavenging Assay (FRSA)

2,2-diphenyl 1-picrylhydrazyl (DPPH) radicals were used in order to evaluate the antioxidant potency of NPs. To a volume of 180 μL of DPPH solution (at a concentration of 2.35 mg/100 mL in methanol), 20 μL of the diluted NPs test sample were added. The stock solutions (4 mg/mL dissolved in DMSO) were further diluted in DMSO to a final volume of 100 µL in order to reach the final concentrations of 7.40, 22.22, 66.66, and 200 μg/mL used for this assay. Optical density at 515 nm was recorded for each test sample with the help of a microplate reader (Platos R, 496. AMP, AMEDA Labordiagnostik GmbH, Graz, Austria) once the incubation (37 °C for 30 min) was completed. To calculate the percent of free radical scavenging assay (FRSA), the following formula was utilized:

% FRSA = (1 − Abs/Abc) × 100

where Abc and Abs are the negative control and sample absorbance respectively. The absorbance of DPPH was 1. The ascorbic acid used in this assay served as the positive control. Values of IC50 calculated for each sample showed significant radical scavenging efficiency. The values were 50% greater and calculated using the two-fold serial dilution method.

### 2.11. α-Amylase Inhibition Assay

The in vitro α-amylase inhibitory activity was determined following the previous standard protocol with slight modifications [43]. Reaction mixture was prepared by mixing 25 μL α-amylase, 10 μL of NPs stock solutions (4 mg/mL in DMSO), 40 μL starch solution in (2 mg/mL) and 15 μL phosphate buffer (pH 6.8). Starch solution was prepared in potassium phosphate buffer. The resulting mixture was subjected to 30 min incubation at 50 °C in the 96-well plate. A total of 20 μL HCl (1 M) was used for halting the reaction, followed by the addition of 90 μL iodine reagent, prepared by mixing 5 mM potassium iodide and 5 mM iodine (in phosphate buffer). The mixture of phosphate buffer with DMSO instead of enzyme and NPs respectively served as the blank (negative control). Acarbose (250 μM) served as the positive control. Absorbance was recorded at 540 nm using a microplate reader (Platos R, 496. AMP, AMEDA Labordiagnostik GmbH, Graz, Austria). The activity was expressed as a percentage of α-amylase inhibition. The following equation was used for calculation:

% α − amylase inhibition = (As − An)/(Ab − An) × 100

where As = sample’s absorbance, An = negative control’s absorbance and Ab = blank’s absorbance.

### 2.12. Brine Shrimp Lethality Assay

Synthesized NPs (20 mg/mL stock in water) were tested for probable lethality in the 96-well plate (300 µL) for 24 h against *Artemia salina* (brine shrimp). Brine shrimp is well-known in study of the toxicological effect of a substance on living organisms [42]. Larvae of *Artemia salina* was used for this purpose following protocols described by [42]. The eggs of brine shrimp were subjected to incubation for a period of 24–48 h for hatching. During the process, constant oxygen supply in sterile sea water (38 g/L) was ensured. Sterile sea water was supplemented with 6 mg/L dried yeast in a specifically designed plastic tray with two-compartments under proper illumination. Illumination provides the necessary light and temperature (30–32 °C) for hatching. A total of 10 mature nauplii (phototropic) were picked using Pasteur pipette and introduced into the wells. The required volume, as described in developed previous protocols, of the test extracts contain 1% DMSO in sea water. The final concentrations of NPs were adjusted to 25, 50, 100, and 200 μg/mL before transferring it to the wells containing shrimp larvae and sea water. A volume of 300 μL was adjusted in each well. Serial concentrations of doxorubicin (ranging from 1 to 10 µg/mL) were taken as the positive control while that of the DMSO (1%) in sea water served as the negative control. After an incubation period of 24 h, live shrimps were quantified and median lethal concentration (LC50) was determined by using the table curve 2D v5.01 of the test extracts with ≥50% mortality. Doxorubicin was used because it is a renowned chemotherapeutic agent used for a variety of cancer treatments [44].

### 2.13. Compatibility with Isolated Human Red Blood Cells (hRBCs)

Hemolytic assay was performed so as to assess the bio-safe nature of the synthesized ZnO NPs against freshly isolated human red blood cells. The fresh blood samples were collected from 2 male and 1 female (average age 28 years) healthy students, having no medical history with sterile syringes. All procedure performed in the study involving human participants were in accordance with the ethical standards of the International and National Research Committees and with the 1964 Helsinki Declaration and its later amendments. Informed consent was obtained from all participants involved in this study. In order to prevent blood clotting, the blood was dispensed in an EDTA tube [45]. To isolate the red blood cells, 1 mL blood was centrifuged at 14,000 rpm for 5 min. About 9.8 mL of PBS (phosphate-buffer saline) (pH: 7.2) was introduced into 200 µL of pelleted erythrocyte, followed by shaking in PBS. Approximately 100 µL of the test NP solution and erythrocyte suspension were added to a 1.5 mL Eppendorf tube. The resulting tubes were then incubated for 1 h at 35 °C followed by centrifugation at 10,000 rpm for 10 min. The supernatant thus obtained was collected and dispensed (100 µL) in a 96-well plate and the release of hemoglobin was assessed using a BioTek ELX800 Absorbance Microplate Reader (BioTek Instruments, Colmar, France) at 540 nm. The positive and negative controls in this study were Triton X-100 and DMSO respectively. The results correspond to the percent hemolysis brought about by NP dilution using the following formula:

% Hemolysis = [Sample_Abs_ − Negative control_Abs_/Positive control_Abs_ − Negative control_Abs_]. × 100

### 2.14. Statistical Analysis

Statistically, all of the results evaluated for assays were evaluated using one-way variance (ANOVA) analysis followed by Duncan’s and Tukey test using PASW Statistics 18. *p* < 0.01, *p* < 0.001 or *p* < 0.05 was deemed significant when appropriate. Data was expressed in terms of mean ± SD.

## 3. Results

ZnO NPs were successfully prepared via different plant sources such as wild plant, in vitro derived plantlets, seeds, and callus cultures by simple co-precipitation. All of the NPs, i.e., seed extract mediated ZnO NPs (S-ZNPs), in vitro derived plantlets extract mediated ZnO NPs (P-ZNPs), wild plant extract mediated ZnO NPs (W-ZNPs) and callus extract mediated ZnO NPs (C-ZNPs) were characterized through various techniques such as powdered XRD, FTIR, SEM and TGA.

### 3.1. XRD Analysis

**Figure 1:**
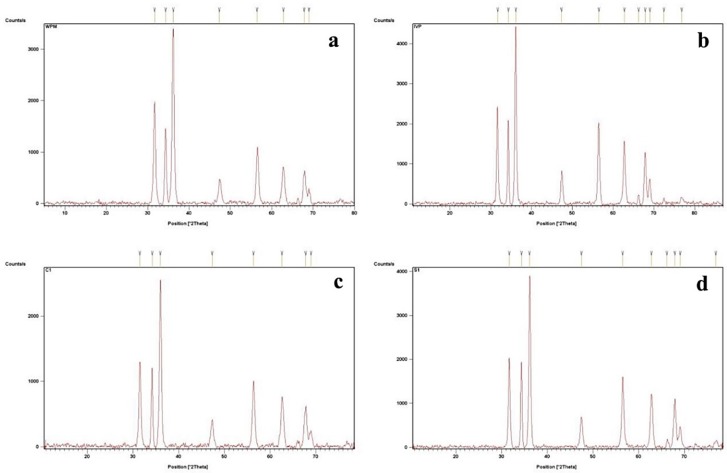
X-ray diffraction analysis of (**a**) wild plant extract mediated ZnO nanoparticles (NPs) (W-ZNPs), (**b**) in vitro derived plantlets extract mediated ZnO NPs (P-ZNPs), (**c**) callus extract mediated ZnO NPs (C-ZNPs) and (**d**) seed extract mediated ZnO NPs (S-ZNPs).

Powder XRD was employed to verify the crystallinity and phase distribution of the synthesized ZnO NPs. Figure 1 shows XRD pattern of W-ZNPs (Figure 1a), P-ZNPs (Figure 1b), C-ZNPs (Figure 1c) and S-ZNPs (Figure 1d). The peaks for W-ZNPs were recorded at 31.6768, 34.2977, 36.2065, 47.3166, 56.4356, 62.7896, 67.8602 and 68.946. The peaks for S-ZNPs were recorded at 31.7122, 34.3859, 36.147, 47.4947, 56.5241, 62.8238, 66.2066, 67.9154, 69.1088 and 76.9091. C-ZNPs showed peaks at 31.5134, 34.2325, 36.0115, 47.3725, 56.3484, 62.5803, 67.8055 and 68.9589. P-ZNPs showed peaks at 31.682, 34.3597, 36.1419, 47.3757, 56.492, 62.7547, 66.2668, 67.8583, 69.0672, 72.438 and 76.8551. The sizes of ZnO NPs calculated using the Scherrer equation were 44.06, 30.8, 44 and 46 nm synthesized respectively for W-ZNPs, P-ZNPs, C-ZNPs and S-ZNPs, respectively.

### 3.2. Scanning Electron Microscopy

**Figure 2:**
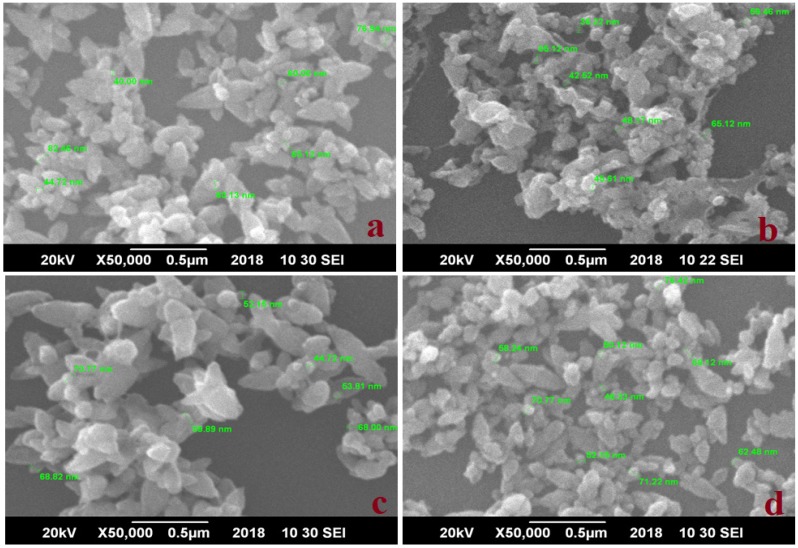
SEM images of (**a**) W-ZNPs, (**b**) P-ZNPs, (**c**) C-ZNPs and (**d**) S-ZNPs.

Size and surface morphology of the prepared ZnO NPs were imaged through SEM analysis. Figure 2 displays SEM images of W-ZNPs (Figure 2a), P-ZNPs (Figure 2b), C-ZNPs (Figure 2c) and S-ZNPs (Figure 2d). Flower-like morphology was observed for W-ZNPs, C-ZNPs and S-ZNPs. In the case of P-ZNPs, small petal-like structures attached to form a flower-shaped morphology with irregular shapes. The average size of ZnO NPs evaluated from SEM analysis were 61.3, 51.7, 64 and 61.9 nm synthesized for W-ZNPs, P-ZNPs, C-ZNPs and S-ZNPs, respectively. Considering the precision of SEM, we observe that these sizes are in a close proximity with those calculated using the Scherrer equation following XRD analysis, and therefore, confirm it.

### 3.3. FT-IR Analysis

**Figure 3:**
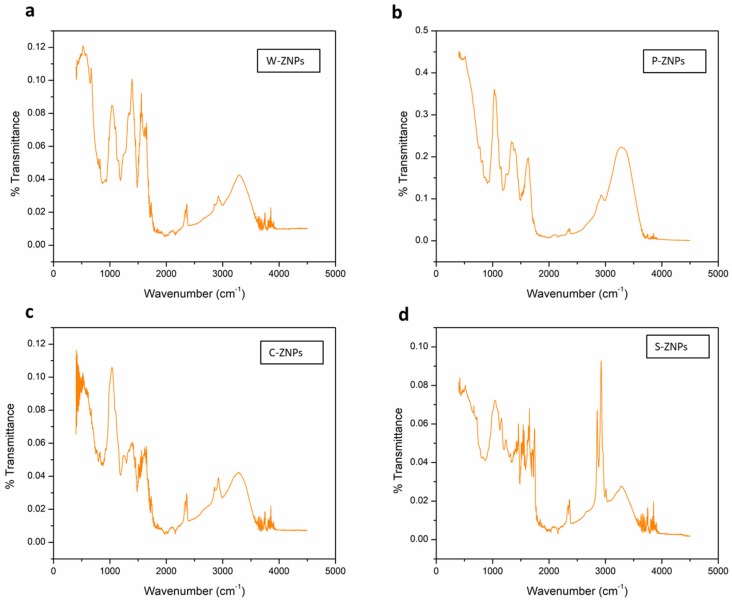
FTIR spectral analysis of (**a**) W-ZNPs, (**b**) P-ZNPs, (**c**) C-ZNPs and (**d**) S-ZNPs.

Fourier transform infrared spectroscopy was performed in order to access the involvement of biological molecules towards the formation of ZnO NPs. The results illustrate significant absorption spectra ranging from 400 to 4000 cm^−1^ (Figure 3). In all four FT-IR spectrums, i.e., W-ZNPs (a), P-ZNPs (b), C-ZNPs (c) and S-ZNPs (d), there is an absorption band at 518, 516, 516 and 516 cm^−1^ which is the characteristic signal of the Zn–O bonding, verifying that the prepared material was indeed ZnO [21]. Moreover, the peaks at 1633 and 1637 cm^−1^ in the case of P-ZNPs and S-ZNPs and peaks at 1336, 1396 and 1400 cm^−1^ in P-ZNPs, W-ZNPs and C-ZNPs were obtained as a result of aromatic rings, and their functional groups exist in the plant sources used [22]. Peaks obtained in the region of 3286 to 3290 and 1051 to 1058 cm^−1^ in all four FTIR spectrums are attributed to C–H and C–O–C stretching [46] and peaks at 2360 cm^−1^ are due to C=C stretching vibrations. These results illustrate the possible role of biological molecules in the fabrication of ZnO NPs.

### 3.4. Thermogravimetric Analysis (TGA)

**Figure 4:**
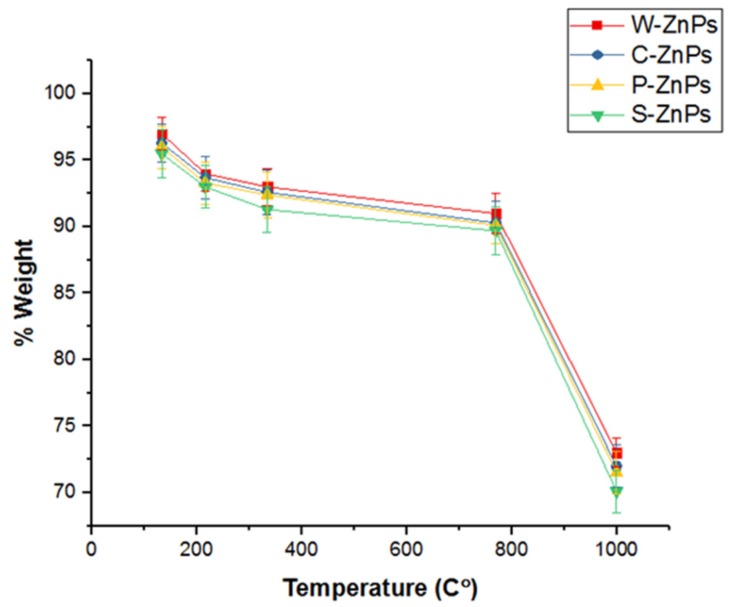
Thermal stability of ZnO NPs at different temperatures. The experiment was carried out in triplicate. The values given in figure are mean ± standard deviation of the original data.

The thermal stability of the green synthesized NPs (20 mg) was evaluated via TGA by subjecting the NPs to a temperature in the range of 0–1100 °C (Figure 4). Initial weight loss in W-ZNPs, C-ZNPs, P-ZNPs and S-ZNPs was observed at 134 °C, 137 °C, 138 °C and 141 °C respectively, which is attributed to the loss of moisture. The second major decomposition of the four samples occurred between 220 °C and 350 °C. The third major weight loss occurred at a high temperature, ≥720 °C. The loss of weight in this event was ~22%, ~23%, ~23% and ~24%, respectively.

### 3.5. Antibacterial Activities

**Figure 5:**
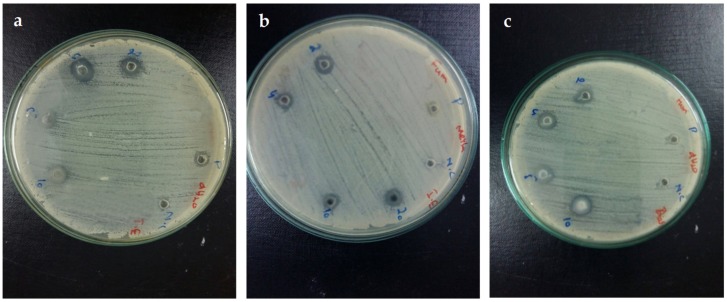
NPs activity against (**a**) *S. aureus*, (**b**) *K. pneumonia*, (**c**) *P. aeruginosa.* The experiments were carried out in triplicate. The values given in Table 1, expressed as mean ± standard deviation of the original data.

Antibacterial assay was performed against both gram-positive and gram-negative bacterial strains (Figure 5). All of the NPs showed considerable antibacterial potency against the tested strains (*S. aureus*, *B. subtilis*, *K. pneumonia*, *P. aeruginosa* and *E. coli*).

Samples showing the zone of inhibition, ≥12 mm, to be considered as significantly active. The data obtained was further processed to appraise MIC through broth micro dilution method. Among all of the tested strains, *S. aureus* was found to be the most susceptible bacterial strain with maximum zone of inhibition against S-ZNPs (20 ± 0.98 mm) with a MIC of ≥100 μg/mL. C-ZNPs proved most potent against *K. pneumoniae* (17 ± 1.11 mm) having a MIC of ≥100 μg/mL. *B. subtilis* and *E. coli* showed maximum susceptibility towards W-ZNPs (9 ± 0.60 mm and 10 ± 0.75 mm, respectively. P-ZNPs proved to be most potent against *P. aeruginosa* showing a maximum zone of inhibition (17 ± 0.78 mm). Overall, S-ZNPs proved to be the most potent antibacterial agents, showing clear zones of inhibition (20 ± 0.98 mm, 8 ± 0.58 mm, 15 ± 0.93 mm, 13 ± 0.54 mm and 7 ± 0.92 mm respectively) against *S. aureus*, *B. subtilis*, *K. pneumonia*, *P. aeruginosa* and *E. coli*. Cefixime served as positive control for *K. Pneumoniae*, *P. aeruginosa* and *E-coli* while Roxithromycin served as positive control for *S. aureus* and *B. subtilus*. DMSO served as negative control during the analysis. Figure 5 shows the zones of inhibition against the bacterial strains, while the measured growth inhibitory zones and MIC of various samples is given in Table 1. These results are in the range of previous work dealing with the antibacterial activity of biosynthesized ZnO NPs [47,48,49,50,51].

### 3.6. Antioxidant In Vitro Capacities

**Figure 6:**
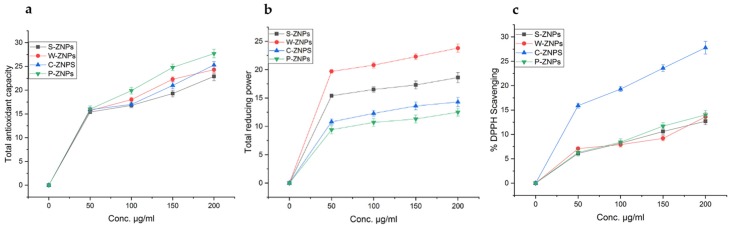
Total antioxidant capacity (TAC) (**a**), total reducing power (TRP) (**b**) and DPPH-free radical scavenging assay (FRSA) (**c**) potential of the biosynthesized ZnO NPs. The experiments were carried out in triplicate. The values given in figure are mean ± standard deviation of the original data.

TAC, DPPH-FRSA and total reducing power (TRP) assays were performed through well-established procedures [52]. The highest TAC was found to be 27.7 ± 0.9 µgAAE/mg for P-ZNPs followed by C-ZNPs, W-ZNPs and S-ZNPs having TAC of 25.3 ± 0.7 µgAAE/mg, 24.3 ± 0.8 µgAAE/mg and 22.9 ± 0.9 µgAAE/mg, respectively (Figure 6a). TRP assay was also performed to further assess the presence of the antioxidant potential of NPs. The method implicates reductones investigation. These reductones are species with antioxidant potency that are believed to be due to their capacity to donate H-atom. This results in discontinuation of free radical chains [5]. Figure 6b shows the TRP of the bio augmented ZnO NPs used at different concentrations. The value of reducing power decreases with the decreased concentration of test samples. The maximum value of reducing power was observed at 200 μg/mL, which was 23.8 ± 0.7 µgAAE/mg for W-ZNPs followed by 18.6 ± 0.9 µgAAE/mg for S-ZNPs, 14.3 ± 0.8 µgAAE/mg for C-ZNPs and 12.5 ± 0.7 µgAAE/mg for P-ZNPs. The least reducing potential of 2.83 ± 1.43 µgAAE/mg, 2.40 ± 2.1 µgAAE/mg, 2.19 ± 1.62 µgAAE/mg, and 2.31 ± 2.02 µgAAE/mg was recorded at 12.5 μg/mL concentration of synthesized NPs. ZnO NPs were subjected to DPPH radical scavenging assay to further confirm the antioxidant potency of biosynthesized ZnO NPs. DPPH assay relies on creation of a light yellow diphenyl picrylhydrazine molecule formed as a result of the reduction of the DPPH moiety after it accepts the electron from a donor species [53]. Moderate DPPH radical scavenging was found at elevated concentrations, i.e., 200 μg/mL, which was 12.7 ± 0.7%, 13.6 ± 0.9%, 27.8 ± 1.3% and 14.0 ± 0.9% for S-ZNPs, W-ZNPs, C-ZNPs and P-ZNPs, while no radical scavenging is testified at lowest concentration (12.5 μg/mL). From the results summarized in Figure 6c, it can be proposed that some of the compounds involved in the stabilization and reduction of NPs during the synthesis process via aqueous extracts of *S. marianum* were of antioxidant nature.

### 3.7. α-Amylase In Vitro Inhibition Potential

**Figure 7:**
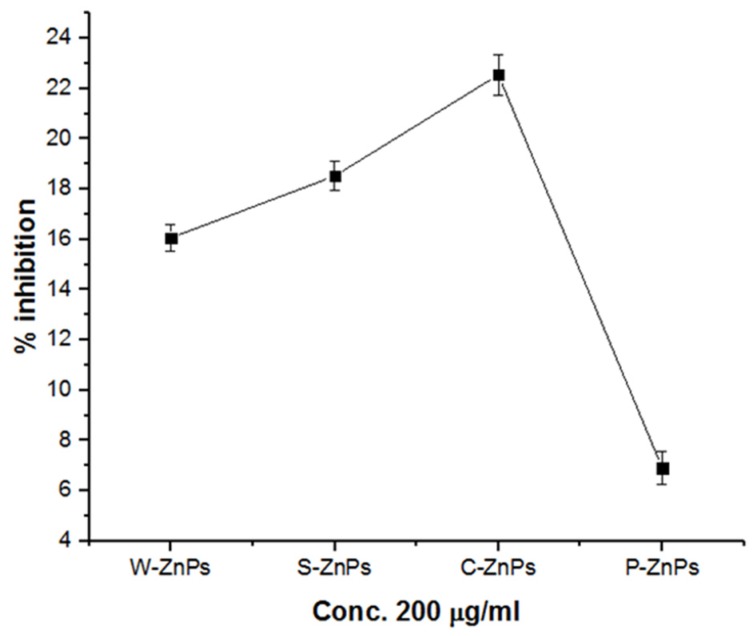
ZnO NPs induced % inhibition of α-amylase. The experiments were carried out in triplicate. The values given in figure are mean ± standard deviation of the original data.

The inhibition of key enzymes, such as α-amylase, which is responsible for hydrolysis of carbohydrates, is an effective strategy to maintain blood glucose levels within the permissible range, which is crucial in human pathologies such as diabetes mellitus [47]. In the current study, the α-amylase inhibition potential of ZnO NPs was evaluated in vitro at moderate concentration (i.e., 200 µg/mL). Percent inhibition of α-amylase is given in Figure 7. Among the particles, C-ZNPs showed the highest inhibition potential of 22% followed by S-ZNPs and W-ZNPs with inhibition at 18% and 16%, respectively. However, P-ZNPs displayed the lowest α-amylase inhibition potential (*ca* 5% amylase inhibition). The different inhibition potentials of the ZnO NPs may be due to their different physicochemical and morphological features resulting from the plethora of reducing and capping agents in the corresponding extracts [54].

### 3.8. Antiproliferative/Cytotoxic Activity against HepG2 Cancer Cells

**Figure 8:**
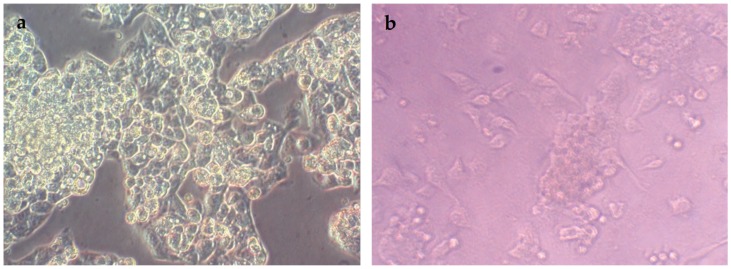
Characteristic pictures of non-treated (**a**) and treated (**b**) NPs activity against HepG2 cell line.

The antiproliferative/cytotoxicity analysis of the synthesized ZnO NPs (20 mg/mL stock in water) against HepG2 cell lines was evaluated using MTT assay. From the results, it is clear that all samples showed high cytotoxicity towards non-treated HepG2 cells. The percent viability of the non-treated cells (NTCs) was observed to be 100.90 ± 5.30% of viable cells, which dropped to 22.88 ± 1.58% of viable cells in the presence of S-ZNPs. Maximum antiproliferative/cytotoxic effect was observed in the presence of W-ZNPs and C-ZNPs (13.16 ± 1.83% and 13.10 ± 2.83% of viable cells respectively) at 100 µg/mL dose. P-ZNPs also showed considerable cytotoxicity (20.12 ± 1.05) against HepG2 cancer cell lines. Figure 8 displays the results of the MTT assay. The results are summarized in Table 2. The lower the values, the higher the effectiveness against liver cancer cells. Our results are similar to previous work [45].

### 3.9. Brine Shrimp Lethality Assay

*Artemia salina* larvae (brine shrimps) were used in order to assess the toxicological effect of samples. Indeed, brine shrimp is a well-known model to study the toxicological effect of a substance on living organisms [42]. Here, we used doxorubicin as a control because it is a renowned chemotherapeutic agent already used for a variety of cancer treatments [44]. All of the samples evaluated for toxic effect against brine shrimps’ larvae were found to be significant. The results of all of the samples screened against *A. salina* larvae are summarized in Table 3. In our hands, doxorubicin during the assay presented an LC_50_ value of 5.92 μg/mL, while among the four NPs samples, the C-ZNPs and P-ZNPs were found to be more toxic showing an LC_50_ of 10.17 μg/mL and 21.06 μg/mL respectively. The concentration of the sample was found to be directly proportional to the degree of lethality. The brine shrimp lethality assay is known to be a suitable tool to evaluate the toxicity of compounds, but it has also been proposed as a good tool to screen pharmacological activities in NPs and their toxicity results can be matched with their reported ethno-pharmacological role. The brine shrimp results were generally interpreted as follows: LC50 < 1.0 μg/mL for highly toxic compounds; LC50 1.0–10.0 μg/mL for toxic compounds; LC50 10.0–30.0 μg/mL for moderately toxic compounds; LC50 30.0–100.0 μg/mL for mildly toxic compounds, and >100.0 μg/mL as non-toxic [42,43]. Here, the samples were considered as mildly to moderately toxic, with C-ZNPs and P-ZNPs being moderately toxic, whereas S-ZNPs and W-ZNPs being mildly toxic. For comparison, the renowned chemotherapeutic agent doxorubicin, used to cure a variety of cancers, was considered as toxic.

### 3.10. Compatibility with Human Red Blood Cells (hRBCs)

**Figure 9:**
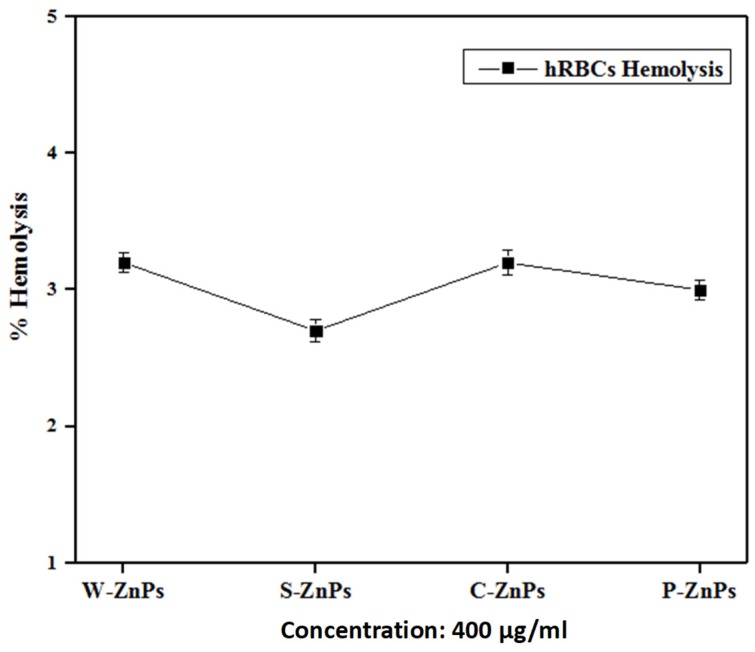
Compatibility of the synthesized ZnO NPs against human red blood cells. The experiments were carried out in triplicates. The values given in figure are mean ± standard deviation of the original data.

To further characterize the bio-safe nature of green synthesized NPs, their compatibility with Human Red Blood Cells (hRBCs) was evaluated. The tested NPs are nontoxic to normal cells only. The results are shown in Figure 9. Based on the standards of “American Society for Testing and Materials Designation” any material having ≥5% hemolysis is considered as hemolytic, slightly hemolytic is 2–5% while nonhemolytic is ≤2% [55]. The hemolysis is measured when the RBC ruptures and the hemoglobin is released upon treatment with NPs (4 mg/mL). In the current study, it was revealed that all of the ZnO NPs synthesized are only slightly hemolytic, even at a high concentration of 400 µg/mL (which is 2-times the concentration used for the toxicological effect evaluation with the brine shrimp lethality assay). The S-ZNPs showed the least hemolytic potency (2.7%) followed by P-ZNPs (3.0%). The other two NPs (C-ZNPs and W-ZNPs) showed hemolytic potency of 3.2%. The results found were in agreement with some other previous reports [56].

## 4. Discussion

In the present study, different types of ZnO NPs were biologically synthesized from wild green parts (W-ZnPs) and seeds (S-ZnPs) or in vitro callus (C-ZnPs) and plantlets (P-ZnPs) of *Silybum marianum*. These synthesized NPs were further characterized and various biological assays were performed. The XRD was performed in order to determine the size of the NPs. In the XRD results, the W-ZnPs (Figure 2a) showed various peaks. Highly stable crystalline NPs with size, calculated using Debye Sherrer’s equation, ranging between 30.8 and 46.0 nm, were obtained from different tissues of *S. marianum*.

The diffractions peaks obtained were in strong agreement with hexagonal geometry of ZnO NPs and in agreement with JCPDS No. 79-2205. The narrow and sharp diffraction peaks demonstrated highly crystalline nature of ZnO NPs. Our results are similar to previous results reported [9,57,58]. The morphological characterization of NPs was done with SEM analysis. The different types of irregular shapes and morphology were noticed, confirming the synthesis of NPs. Our results are similar with previous work [9,59]. The NPs were then checked for stability on high heat using thermogravimetric analyzer. The synthesized NPs breakdown was observed at various temperature starting from 134 °C to 720 °C. The most stable and heat resistant was S-ZnPs at high temperature. The least stable and resistant was W-ZnPs. The stability of these NPs might be due to the capping agents of *S. marianum*’s various components, such as flavonolignans [60]. The current results are also in harmony with previous reports [61,62,63,64].

Following these characterizations, the biological potential of these green bio-assisted synthesized ZnO NPs were evaluated for various potential pharmaceutical applications. The antibacterial potential of these NPs revealed the highest and lowest antibacterial zones of 20.55 ± 0.98 and 7.06 ± 0.92 mm was measured in the case of S-ZnPs against S. *aureus* and *E. coli* respectively. The enhanced antibacterial activity was due to the high surface to volume ratio, that has previously been proposed [31,58,65,66,67]. The NPs were further evaluated for various antioxidant mechanisms: the highest total antioxidant capacity was measured for P-ZNPs while the lowest was noted for S-ZnPs; the highest free radical scavenging activity was observed for C-ZnPs and the lowest for S-ZnPs; and finally, the highest total reducing power value was recorded for W-ZNPs while the lowest was for P-ZNPs. These observations match with previous works reporting the high antioxidant potential of plant-based NPs [68,69]. The in vitro α-amylase inhibition assay was performed for all NPs. This key enzyme hydrolyses α bonds of polysaccharides, such as starch and glycogen, yielding glucose and maltose [47]. Reducing its activity through reversible enzymatic inhibition could be an effective strategy to maintain blood glucose level within the permissible range, which is crucial in human pathologies such as diabetes mellitus [47]. Here, the ZnO NPs showed a moderate in vitro α-amylase inhibition that could be relevant for this form of anti-diabetes strategy. The flavonoids and polyphenols capping of NPs could be involved in the antibacterial, antioxidant and α-amylase inhibition potentials of plant-based NPs [70,71]. With *S. marianum* being a rich source of these compounds, in particular of flavonolignans [60], our results could be in agreement with these previous works. The evaluation of their antiproliferative/cytotoxic activity performed against human hepato-cellular carcinoma cells HepG2 showed the great potential of these green ZnO NPs. This was the case, especially when considering their mild to moderate toxicity revealed by the brine shrimp lethality assay and their bio-safe nature assessed by non-hemolytic against human red blood cells. Further studies are needed to confirm these in vitro assays and characterized the molecular mechanism behind these biological activities, but our present results already sheds light on the great biomedical potential of plant-based NPs, in good agreement with previous reports [72,73,74].

## 5. Conclusions

NPs have become an indispensable tool for research in the current era owing to their wide array of applications in almost every field. The most widely explored applications, however, are in the field of medical sciences therefore, the current study involves exploitation of NPs synthesized from various parts of *Silybum marianum* (L.) Gaernt. (Milk thistle) as an antibacterial, antiproliferative/cytotoxic against cancer cells, α-amylase inhibitory and antioxidant agents. ZnO NPs were selected for the current study owing to their bio-compatible nature and efficient synthesis protocols. All of the NPs synthesized during the current study showed potent biological activities. S-ZNPs proved to be the most potent antibacterial agent against the tested bacterial species. S-ZNPs were also found to have the most potent antioxidant capacity. C-ZNPs showed a significant antiproliferative/cytotoxic activity against HepG2 human cancer cells. However, C-ZNPs were found to be the most toxic. Nevertheless, these NPs were only mildly to moderately toxic, as revealed by the brine shrimp lethality assay and their bio-safe nature, and hRBC compatibility was assessed by their non-hemolytic action against human red blood cells. These results demonstrated that green ZnO NPs bio-synthesized using *S. marianum* plant extracts could be suitable candidates for various future applications in biomedical research.

## Figures and Tables

**Table 1:** Zones of inhibition (expressed in mm) and minimum inhibitory concentrations (MICs) (expressed in µg/mL) of the synthesized NPs against various bacterial strains.

Samples	*S. aureus*		*B. subtilis*		*K. pneumonia*		*P. aeruginosa*		*E. coli*	
C-ZNPs	16.03 ± 0.58 ^ab^	33.33	7.48 ± 0.60 ^ab^	−	17.66 ± 1.11 ^a^	≥100	16.28 ± 0.33 ^a^	≥100	9.83 ± 0.47 ^a^	−
S-ZNPs	20.55 ± 0.98 ^a^	≥100	8.29 ± 0.58 ^a^	−	15.56 ± 0.93 ^ab^	−	13.88 ± 0.54 ^b^	−	7.06 ± 0.92 ^ab^	−
W-ZNPs	14.85 ± 1 ^b^	100	9.58 ± 0.60 ^a^	−	15.39 ± 0.86 ^ab^	−	15.53 ± 0.66 ^ab^	−	1.07 ± 0.75 ^a^	−
P-ZNPs	15.96 ± 0.88 ^b^	≥100	8.21 ± 0.97 ^a^	−	13.44 ± 0.79 ^b^	≥100	17.91 ± 0.78 ^a^	≥100	1.73 ± 0.48	−
positive control	13.74 ± 0.7 ^bc^		8.06 ± 0.7 ^a^		12.32 ± 0.9 ^b^		12.85 ± 78 ^b^		8.33 ± 0.7 ^ab^	
negative control	8.77 ± 0.6 ^cd^		6.65 ± 0.5 ^ab^		7.03 ± 0.6 ^c^		7.91 ± 0.5 ^c^		7.85 ± 0.6 ^ab^	

Data is expressed in terms of mean ± SD. Different letters indicate significant differences between conditions (*p* < 0.05).

**Table 2:** Viability of human hepatocellular carcinoma (HepG2) cells treated with the different NPs.

Sample	% Viability (Mean ± SD)
NTC	100.90 ± 5.30 ^c^
W-ZNPs	13.16 ± 1.83 ^a^
C-ZNPs	13.10 ± 2.83 ^a^
S-ZNPs	22.88 ± 1.58 ^ab^
P-ZNPs	20.12 ± 1.05 ^ab^

Sample concentration: 1% of the stock provided (200 µg/mL for 20 mg/mL stock), NTC = Non-treated HepG2 cells; SD = Standard deviation. The experiments were carried out in triplicate. The values given in the table are mean ± standard deviation of the original data. Different letters indicate significant differences between conditions (*p* < 0.05).

**Table 3:** Lethality of the synthesized ZnO NPs against brine shrimp.

Sample	% Mortality Rate (at 200 µg/mL NPs)	LC 50 (μg/mL)
C-ZNPs	96.6 ± 0.6 ^a^	10.17
S-ZNPs	90 ± 0.6 ^b^	62.5
W-ZNPs	93.3 ± 0.6 ^ab^	38.46
P-ZNPs	96.6 ± 0.6 ^a^	21.06

The experiments were carried out in triplicates. The values given in table are mean ± standard deviation of the original data. Different letters indicate significant differences between conditions (*p* < 0.05).
